# Transcriptomic and Metabolomic Analyses of *Diaphorina citri* Kuwayama Infected and Non-infected With *Candidatus* Liberibacter Asiaticus

**DOI:** 10.3389/fphys.2020.630037

**Published:** 2021-02-24

**Authors:** Kai Liu, Jiawei He, Ziying Guan, Mingzhao Zhong, Rui Pang, Qunxin Han

**Affiliations:** ^1^College of Agriculture and Biology, Innovative Institute for Plant Health, Zhongkai University of Agriculture and Engineering, Guangzhou, China; ^2^Guangdong Provincial Key Laboratory of Microbial Safety and Health, State Key Laboratory of Applied Microbiology Southern China, Guangdong Institute of Microbiology, Guangdong Academy of Sciences, Guangzhou, China

**Keywords:** *Diaphorina citri*, *Candidatus* Liberibacter asiaticus, interaction, transcriptome, metabolome

## Abstract

The Asian citrus psyllid *Diaphorina citri* is the transmission vector of Huanglongbing (HLB), a devastating disease of citrus plants. The bacterium “*Candidatus* Liberibacter asiaticus” (*C*Las) associated with HLB is transmitted between host plants by *D. citri* in a circulative manner. Understanding the interaction between *C*Las and its insect vector is key for protecting citrus cultivation from HLB damage. Here, we used RNA sequencing and liquid chromatography-mass spectrometry (LC-MS) to analyze the transcriptome and metabolome of *D. citri* interacting with *C*Las. We identified 662 upregulated and 532 downregulated genes in *C*Las-infected insects. These genes were enriched in pathways involving carbohydrate metabolism, the insects’ immune system, and metabolism of cofactors and vitamins. We also detected 105 differential metabolites between *C*Las-infected and non-infected insects, including multiple nucleosides and lipid-related molecules. The integrated analysis revealed nine pathways—including those of the glycine, serine, threonine, and purine metabolism—affected by the differentially expressed genes from both groups. The network for these pathways was subsequently constructed. Our results thus provide insights regarding the cross-talk between the transcriptomic and metabolomic changes in *D. citri* in response to *C*Las infection, as well as information on the pathways and genes/metabolites related to the *C*Las–*D. citri* interaction.

## Introduction

The Asian citrus psyllid *Diaphorina citri* Kuwayama (Hemiptera: Psyllidae) is the main citrus pest, and it is the most widely distributed insect vector of huanglongbing (HLB, or citrus greening disease). HLB is a serious threat for the citrus industry worldwide (Bové, 2006; Gottwald, 2010; Wang and Trivedi, 2013) as it shortens plant life, sharply reduces fruit yield, and may result in plant death. HLB is present in most citrus-producing countries of Asia, Africa, and America (Graca, 1991, 2008). More than 20% of citrus trees in Brazil and 90% in Florida are currently destroyed by HLB, and HLB is currently the most serious infectious disease affecting global citrus yield (Martínez et al., 2009; Ferrarezi et al., 2020).

HLB is associated with the gram-negative bacterium, *Candidatus* Liberibacter asiaticus (*C*Las). *Diaphorina citri* typically feeds on plants infected with *C*Las for some time and then migrates to healthy plants; this accelerates the spread of *C*Las (Mann et al., 2012). Feeding on diseased plants has been shown to increase the fecundity and shorten nymph development duration in *D. citri* (Pelz-Stelinski et al., 2010; Pelz-Stelinski and Killiny, 2016). A study using an electrical penetration graph showed that the salivary time of *D. citri* adults feeding on diseased plants was significantly prolonged compared to that of *D. citri* not feeding on diseased plants (Cen et al., 2012). Moreover, infected *D. citri* have been shown to migrate to healthy plant earlier and have the ability to fly father than uninfected ones, and as bacterial contents increased, infected females became more attractive to males (Martini et al., 2015; Ren et al., 2016). *C*Las infection downregulated the gene expression level of a protease and its transporter complex in *D. citri* nymphs, and the immune system’s ability to synthesize and release immune-related proteases was decreased, allowing the reproduction of *C*Las (Tiwari et al., 2011). In addition, *C*Las can affect the behavior, gene expression, and substance metabolism of *D. citri*, thus promoting its transmission and spread. However, the genetic mechanisms regulating these phenomena are largely unknown.

The draft genome sequence of *D. citri* is available in the National Center for Biotechnology Information (NCBI). Accordingly, recent studies have continuously provided more information on the transcriptomic and proteomic changes regarding the species’ hemolymph and midgut (Vyas et al., 2015; Gill et al., 2017; Kruse et al., 2017, 2018; Yu et al., 2020), revealing the effects of *C*Las infection on *D. citri*’s nutrition metabolism, nymphal development, and immunity. Most aforementioned previous studies have focused on specific tissues of *D. citri* due to their implication in the insect’s immune system. In the present study, we compared the whole-body transcriptomes and metabolomes between *C*Las-infected and non-infected *D. citri*, and we analyzed the DEGs and differentially expressed metabolites to better understand the interaction between *C*Las and *D. citri*.

## Materials and Methods

### Insect Material

The *D. citri* specimens used in this study were collected from Sun Yat-sen University, Guangzhou, China, in 2014. The strain was reared on *Citrus reticulata* Blanco cv. Shatangju in a greenhouse under a 14 h: 10 h light: dark cycle at 26 ± 1°C and 65–70% relative humidity for more than 25 generations. The newly emerged female adults were fed for 14 days on the citrus plants infected with HLB, and they were confirmed to carry *C*Las through sampling detection. Meanwhile, the female adults that was continuously fed on healthy citrus plants for 14 days as control.

### Quantitative PCR Detection of *C*Las

Total RNA (1 μg) was reverse-transcribed into first-strand cDNA using a PrimeScript^TM^ RT reagent kit (Takara Bio, Inc., Otsu, Shiga, Japan). Quantitative PCR (qPCR) was performed using a 10 μL reaction containing 1 μL cDNA, 0.3 μL each of 10 μmol⋅L^–1^ forward and reverse primers (Supplementary Table S1), and 5 μL SYBR FAST Universal qPCR mix (KAPA Biosystems, Woburn, MA, United States) in a LightCycler 480 system (Roche Diagnostics GmbH, Mannheim, Germany), with the following amplification conditions: 5 min at 95°C, followed by 45 cycles at 95°C for 10 s, 60°C for 20 s, and 72°C for 20 s. The qPCR experiments were performed for each sample using three biological and three technical replicates. The expression levels of selected genes were normalized to the expression levels of *D. citri* actin-1. The differential gene expression was calculated using the 2^–△^^△^^*Ct*^ method (Livak and Schmittgen, 2001), and the results were expressed as mean ± SE. The differences in gene expression levels between infected and uninfected *D. citri* were analyzed using *t*-tests in the SPSS 18.0 statistical software (*P* < 0.01).

### RNA-Seq and Differential Expression Analysis

All 20 insect individuals were pooled and ground in liquid nitrogen. Total RNA was extracted using a total RNA extract kit (Omega, Norcross, GA, United States) according to the manufacturer’s instructions. The cDNA libraries from triplicates of each insect line were generated using the Illumina TruSeq Stranded mRNA kit (Illumina, San Diego, CA, Untied States) and were size-selected to an average fragment size of 450 bp. The libraries were sequenced on the Illumina HiSeq Xten platform producing paired-end (PE) 150 bp reads.

Low quality reads from Illumina sequencing were filtered out using Cutadapt software v1.16 with parameters of—discard-trimmed-n3-e0.1 (Martin, 2011). The obtained clean reads were mapped to the *D. citri* genome (NCBI accession No. GCA_000475195.1) with the Hisat2 software (Kim et al., 2015). Read counts for all genes were extracted with HTSeq-count (Anders et al., 2015) and normalized using the R package DESeq2 (Love et al., 2014). The differentially expressed genes (DEGs) between the *C*Las-infected and non-infected lines were estimated by DESeq2 according to the threshold of | log2 ratio| > 1 and adjusted *P* < 0.05 (BH adjustment). The Blast2GO software was used to generate GO annotation of the DEGs. The DEGs were further annotated in the Kyoto Encyclopedia of Genes and Genomes (KEGG) database by KEGG Automatic Annotation Server. The GO functional enrichment and KEGG pathway enrichment of the DEGs were analyzed using the Fisher’s exact test with Benjamini and Hochberg (BH) adjustment.

### Validation of DEGs by qPCR

Twelve genes were selected for qPCR validation based on the differentially expressed level and specific functions (Supplementary Table S1). Following the user manual, the first cDNA strand fragments were synthesized from total RNA using the PrimeScript^TM^ RT Master Mix kit (Takara, Japan). Specific primers for selected genes are listed in Supplementary Table S1. qRT-PCR was performed on an ABI7500 real-time fluorescent quantitative PCR instrument (Applied Biosystems, Foster City, United States). The relative mRNA levels of the selected genes were estimated according to the aforementioned method.

### Metabolite Extraction and LC-MS/MS Analysis

For metabolite extraction, 20 female adults from each insect line were pooled and surface washes of sterile water for 1 min were performed three times. Then the samples were ground to a powder form with an electronic grinding machine. A total of 20 mg of ground sample was placed in an EP tube, and a 1,000 μL extract solution (acetonitrile: methanol: water = 2: 2: 1, with isotopically labelled internal standard mixture) was added. After 30 s of vortex mixing, the samples were homogenized at 35 Hz for 4 min and sonicated for 5 min in ice-water bath. The homogenization and sonication cycle were repeated three times. Then, the samples were incubated for 1 h at −40°C and centrifuged at 12,000 rpm for 15 min at 4°C. The resulting supernatant was transferred to a fresh glass vial for analysis. The quality control sample was prepared by mixing an equal aliquot of supernatant from all the samples. Six biological replications were performed for each insect line.

LC-MS/MS analyses were performed using an UHPLC system (Vanquish, Thermo Fisher Scientific) with a UPLC BEH Amide column (2.1 mm × 100 mm, 1.7 μm) coupled to a Q Exactive HFX mass spectrometer (Orbitrap MS, Thermo Fisher Scientific). The mobile phase consisted of 25 mmol/L ammonium acetate and 25 ammonia hydroxide in water (pH = 9.75) (A) and acetonitrile (B). The analysis was carried with elution gradient as follows: 0–0.5 min, 95% B; 0.5–7.0 min, 95–65% B; 7.0–8.0 min, 65–40% B; 8.0–9.0 min, 40% B; 9.0–9.1 min, 40–95% B; 9.1–12.0 min, 95% B. Column temperature was 30°C. Auto-sampler temperature was 4°C, and the injection volume was 2 μL. The QE HFX mass spectrometer was used for its ability to acquire MS/MS spectra on information-dependent acquisition mode in the control of the acquisition software (Xcalibur, Thermo Fisher Scientific). In this mode, the acquisition software continuously evaluates the full scan MS spectrum. The electrospray ionization (ESI) source conditions were set as follows: sheath gas flow rate 50 Arb, Aux gas flow rate 10 Arb, capillary temperature 320°C, full MS resolution 60,000, MS/MS resolution 7,500, collision energy 10/30/60 in NCE mode, spray voltage 3.5 kV (positive) or −3.2 kV (negative).

### Metabolome Data Processing and Annotation

For peak detection, extraction, alignment, and integration, the raw MS/MS spectra data were converted to the mzXML format using ProteoWizard and processed with an in-house program, developed using R and based on XCMS. Then, an in-house MS2 database (BiotreeDB) was used for metabolite annotation, with a cutoff value of 0.3.

The difference in metabolite contents between the two groups was calculated using analysis of variance (ANOVA) and the Mann-Whitney U-test in SPSS 13.0. Statistical significance was set at *P* < 0.05. MetaboAnalyst^1^ was further used for the hierarchical cluster analysis of all samples and overlapped metabolites. PCA and partial least squares-discriminant analysis (PLS-DA) were conducted to investigate the relationships among the test samples. For the pathway enrichment analysis, the differential metabolites between tested groups were assigned to metabolic pathways using the tool MetPA in MetaboAnalyst. The −log(*P*) and impact values of each metabolic pathway were calculated using the hypergeometric test.

### Integrated Analysis

The transcriptome and metabolome pathways that overlapped were considered, and the corresponding KEGG xml files (KGML) were downloaded from the KEGG pathway database. The interactions among these pathways were estimated using a custom Perl script and the KGML files as input files (Chen et al., 2016; Pang et al., 2018). Finally, the pathway–pathway network was visualized using the Cytoscape software (V.3.6.0).

## Results

### *C*Las-Infected *D. citri*

**FIGURE 1:**
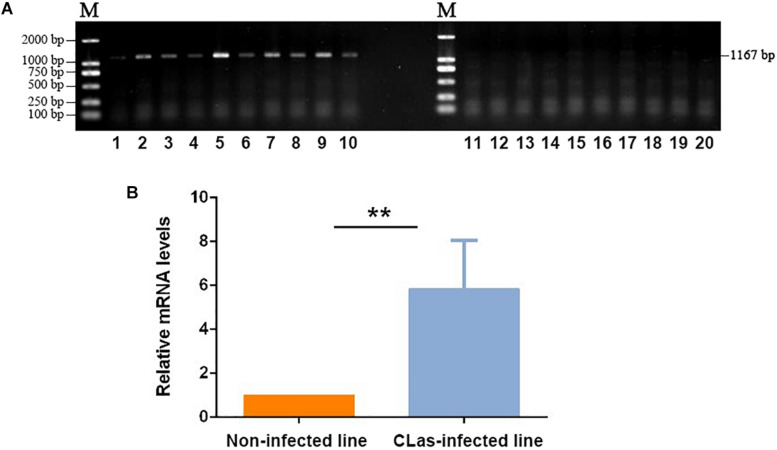
Validation of *C*Las infection in *D. citri* individuals. **(A)** PCR detection of *C*Las-specific sequence in infected and non-infected insect lines. 1–10: Non-infected line; 11–20: *C*Las-infected line. **(B)** qPCR validation of the relative mRNA levels of *C*Las-specific sequences in infected and non-infected insect lines. The data represent mean + SEM (*n* = 3), **P < 0.01 (*t*-test).

The *C*Las-specific fragments had 1,167 bp, and the gel imaging results showed that *D. citri* carried *C*Las after feeding on *C*Las-infected plants for 14 days (Figure 1A). The qPCR test also confirmed the *C*Las infection in the infected insect line, which showed a much higher level of *C*Las mRNA than the control non-infected line (Figure 1B). Thus, the target insect lines could be used for the subsequent transcriptomic and metabolomic analyses.

### Differences in the Transcriptomic Profile Between *C*Las-Infected and Non-infected Insects

The RNA-seq experiments yielded an average 42,029,244 raw paired-end reads (range from 39,283,042 to 44,690,980) for each sample with an average Q30 value of 93.94% (Supplementary Table S2). After quality filtering, we obtained an average 39,538,101 high-quality clean reads for each sample. The average mapping rate of the clean reads to the *D. citri* genome was 81.91% (range from 81.10 to 82.66%), and the mapped reads were used for subsequent analysis.

**FIGURE 2:**
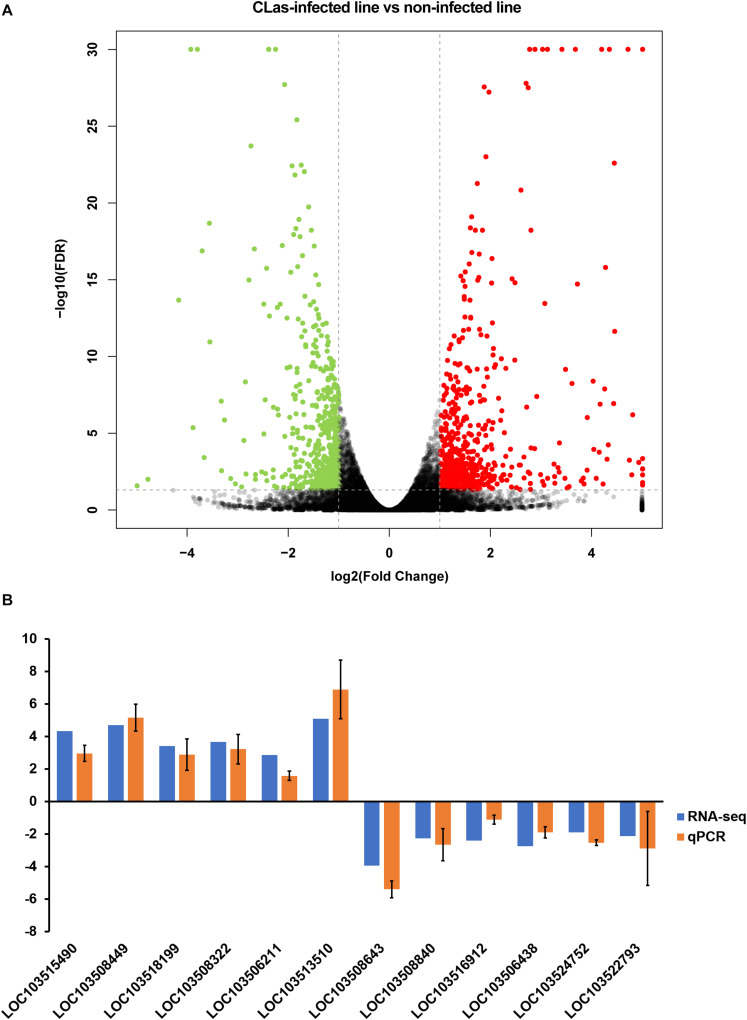
Identification of differentially expressed genes (DEGs) between *C*Las-infected and non-infected *D. citri*. **(A)** Volcano plot for the expression pattern of each gene. Black, red, and green points represent no difference in expression, upregulated genes, and downregulated genes, respectively. **(B)** Comparison of the transcriptome analysis and qRT-qPCR validation. The data represent mean + SEM (*n* = 3).

The correlation test and principal component analysis (PCA) results were consistent regarding the triplicate RNA sequencing (RNA-seq) datasets of *C*Las-infected and non-infected insect lines (Supplementary Figure S1). The comparative analysis revealed that *C*Las infection resulted in 1,194 DEGs (4.97% of the total detected genes in the *D. citri* genome), including 662 up- and 532 downregulated genes (Figure 2A and Supplementary Table S3). Twelve randomly selected DEGs were validated by qPCR (Figure 2B), suggesting the reliability of the RNA-seq results. Among the upregulated genes, we found eight vitellogenin genes and two hemocyanin genes (Supplementary Table S2); the upregulation of these proteins has been observed in previous proteomic studies (Ramsey et al., 2017; Kruse et al., 2018), and our findings further confirmed this at a transcriptomic level.

**FIGURE 3:**
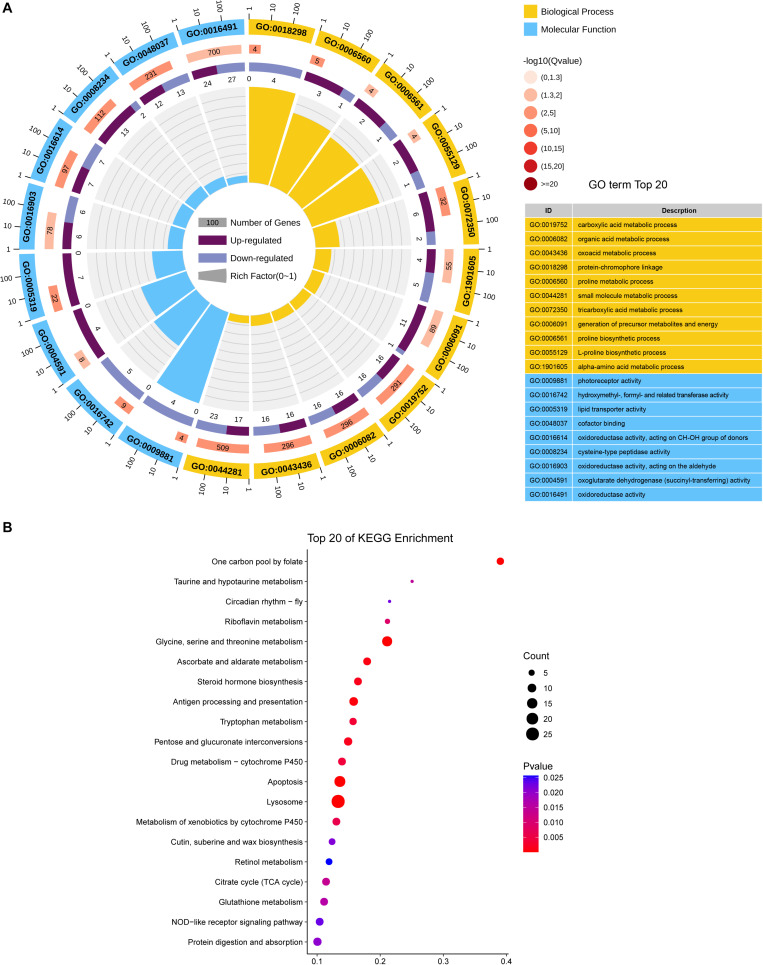
Functional *C*Lassification of DEGs between *C*Las-infected and non-infected *D. citri*. **(A)** GO enrichment analysis of the DEGs. The outer ring represents the number of the top 20 enriched GO terms, and different colors indicate different ontologies. Ring 2 represents the gene counts in the whole genome background, and the color changes according to the *Q*-value of the enrichment analysis. Ring 3 represents the counts for upregulated and downregulated genes in corresponding GO terms. Ring 4 indicates the rich factor (the number of DEGs divided by the number of background genes in the corresponding term). **(B)** Enriched pathways annotated in the KEGG database of DEGs.

DEGs were mostly enriched in gene ontology (GO) functional categories of carboxylic acid metabolic process, organic acid metabolic process, and cofactor binding (Figure 3A and Supplementary Table S3). However, the functional enrichment of upregulated and downregulated genes was largely different. For example, the downregulated genes were mostly related to functions of photoreceptor activity, protein-chromophore linkage, and hydroxymethyl-, formyl-, and related transferase activity, whereas the upregulated genes were preferentially enriched in the functions of lipid transporter activity and lipid transporter activity (Supplementary Table S4).

We further analyzed the pathways affected by the DEGs. The analysis showed the enrichment of DEGs in multiple carbohydrate metabolism pathways, including the citrate cycle (TCA cycle), ascorbate and aldarate metabolism, pentose, and glucuronate interconversions, and pyruvate metabolism (Figure 3B and Supplementary Table S5). This indicated that *C*Las infection may affect the carbon utilization of *D. citri*. Moreover, two immune system-related pathways, antigen processing, and presentation and NOD-like receptor signaling pathway, were found to be significantly enriched with DEGs, suggesting that *C*Las inevitably modulated *D. citri* immunity. Additionally, we noticed that several cofactor and vitamin metabolism pathways were significantly affected by the DEGs. This is in accordance with the GO enrichment in the functional category of cofactor binding. The affected cofactors and vitamins included folate, riboflavin, and retinol.

### Metabolic Differences Conferred by *C*Las Infection

**FIGURE 4:**
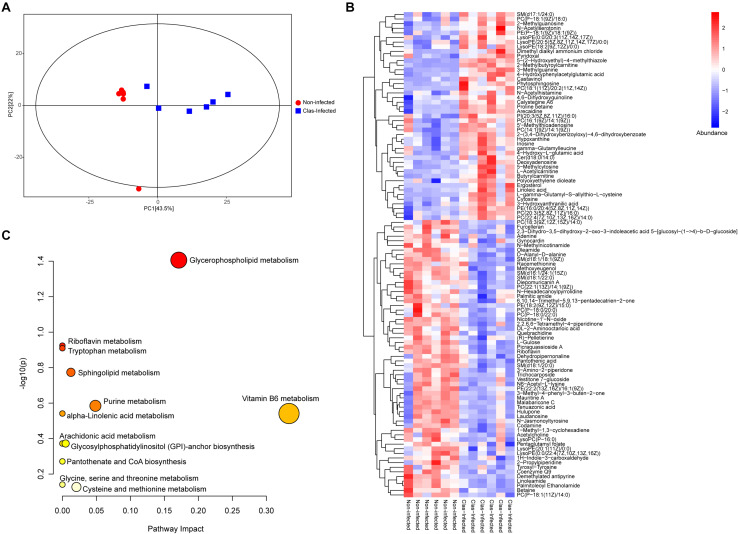
Impact of *C*Las infection on the metabolic profile of *D. citri*. **(A)** Principal component analysis (PCA) plots of the metabolite composition of samples from *C*Las-infected and non-infected *D. citri*. Each symbol represents a sample, different symbol shapes denote different groups. The circular line indicates the 95% confidence interval (Hotelling’s T-squared ellipse). **(B)** Hierarchical cluster and heatmap displaying the abundance levels of the differential metabolites in each sample. **(C)** KEGG pathway annotation of differential metabolites between *C*Las-infected and non-infected *D. citri*. The point size represents the significant compound number in the corresponding pathway.

The metabolome of insects from *C*Las-infected and non-infected lines was measured using LC-MS/MS, which identified a total of 315 metabolites across all samples (Supplementary Table S6). The PCA showed that almost all samples were located within the 95% confidence interval, and these samples were distinctly separated into two groups according to their grouping situation (Figure 4A). The substantial difference between *C*Las-infected and non-infected insect lines was further confirmed by OPLS-DA score plots (Supplementary Figure S2). Of all detected metabolites, 105 showed significant difference in abundance between *C*Las-infected and non-infected lines (Mann-Whitney U-test, *P* < 0.05, Figure 4B). This result revealed that infection with *C*Las led to higher level of steroids (ergosterol), flavonoids (castavinol), and nucleosides such as cytosine, inosine, 5′-methylthioadenosine and 2-methylguanosine. In addition, we observed the upregulation of fatty acid molecules (linoleic acid, polyoxyethylene dioleate, 2-methylbutyroylcarnitine, L-acetylcarnitine, butyrylcarnitine, and polyoxyethylene dioleate) but the downregulation of fatty amides (linoleamide, palmitic amide, and oleamide) in *C*Las-infected insects (Figure 4B).

The metabolic pathway analysis revealed that the pathways of vitamin B6 metabolism, glycerophospholipid metabolism, and purine metabolism were largely affected by the differentially expressed metabolites under *C*Las infection (Figure 4C and Supplementary Table S7). Notably, most of the detected metabolites in these pathways were significantly upregulated, indicating the potentially positive effect of *C*Las in the metabolism of these compounds.

### Regulatory Network Associated With *C*Las Infection

**FIGURE 5:**
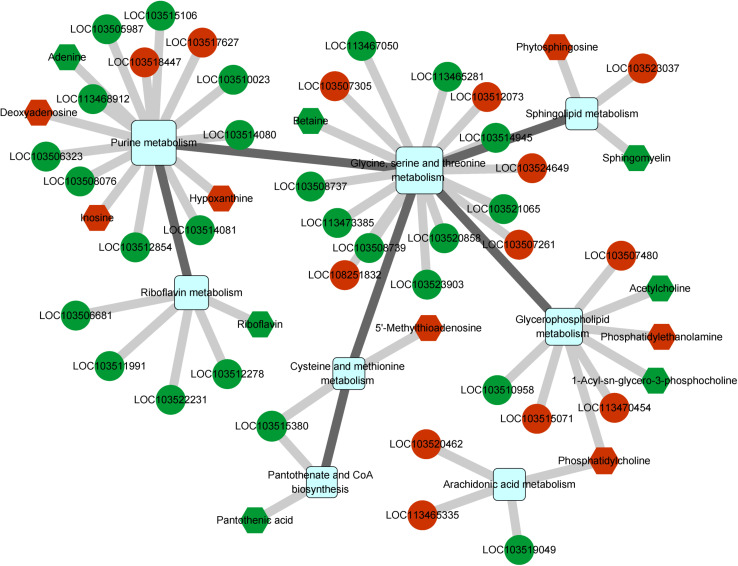
Integrated analysis of regulated pathways identified by transcriptomic and metabolomic analyses. Squares, circles, and hexagons represent pathways, DEGs, and differential metabolites, respectively. For DEGs and differential metabolites, red and green indicate the upregulated and downregulated factors, respectively.

We further determined the gene regulatory network associated with *C*Las infection using an integrated analysis of the transcriptome and metabolome. Nine pathways were found to be affected by both differentially expressed genes and metabolites (Supplementary Table S8), and these pathways could be linked to each other through differential factors except the tryptophan metabolism pathway (Figure 5). The glycine, serine, and threonine metabolism contained the most differential factors, and this pathway interacted with those of the cysteine and methionine metabolism, glycerophospholipid metabolism, sphingolipid metabolism, and purine metabolism. The purine metabolism pathway also harbored multiple differential factors, most of which were significantly upregulated under *C*Las infection.

Among all related differential factors, the downregulated gene LOC103515380 (branched-chain-amino-acid aminotransferase) was involved in both pantothenate and CoA biosynthesis and cysteine and methionine metabolism pathways, indicating its reduced function in the regulatory network under *C*Las infection. In addition, we observed increased levels of the metabolite phosphatidylcholine, an intermediate product of the glycerophospholipid metabolism and the origin of the arachidonic acid metabolism (Supplementary Figure S3). Overall, the network analysis showed that *C*Las infection mainly impacted the metabolism of amino acids, lipids, and cofactors of *D. citri*.

## Discussion

As a key vector of *C*Las transmission, *D. citri* interacts with *C*Las in a circulative and propagative manner; moreover, *C*Las can be distributed in various insect tissues, including the hemolymph and salivary glands (Ammar et al., 2011; Ghanim et al., 2017). Previous studies on the transcriptomic and proteomic changes in *C*Las-infected and non-infected *D. citri* have indicated the involvement of *C*Las infection in the nutrition metabolism, nymphal development, and immunity of *D. citri* (Vyas et al., 2015; Gill et al., 2017; Kruse et al., 2017, 2018; Yu et al., 2020). Here, we focused on the transcriptomic changes in the metabolism pathways conferred by *C*Las infection in *D. citri* by using a combined transcriptomic and metabolic approach. The transcriptomic analysis revealed 662 up- and 532 downregulated genes. The DEG pattern was similar to that previously obtained from the gut transcriptome (Kruse et al., 2017; Yu et al., 2020). The LC-MS/MS analysis identified 315 metabolites in *D. citri*, a number much larger than those obtained in previous GC-MS analyses (Killiny and Jones, 2018). These differential factors represent the body-wide transcriptomic and metabolomic responses to *C*Las infection, which should be compared to the values from specific tissues.

Previous transcriptomic and proteomic analyses of *D. citri* showed an upregulation of transcripts and proteins involved in defense and immunity (including phenoloxidase, vitellogenin, and hemocyanin) in response to *C*Las infection (Kruse et al., 2017, 2018; Ramsey et al., 2017). Our data further showed that genes in the pathways of antigen processing and presentation and NOD-like receptor signaling were significantly affected (Supplementary Table S5). The heat shock proteins (HSP) coding genes in these two pathways were upregulated, as reported by Kruse et al. (2017) and Yu et al. (2020). HSPs have been recognized as important immune response factors to biological and environmental stresses in multiple insect systems (Pockley, 2003; Aguilar et al., 2005; Gotz et al., 2012). Thus, the upregulation of HSPs indicated the activation of *D. citri*’s immune system conferred by *C*Las infection. The draft genome sequence indicated that *D. citri* presented the Toll immune signaling pathway, as shown by previous studies (Ramsey et al., 2015; Kruse et al., 2017; Yu et al., 2020). In the transcriptome herein obtained, we also observed the upregulation of Ras-related C3 botulinum toxin substrate (LOC103512003), a gene induced by Toll-like receptor 2 in the Toll immune signaling pathway (Arbibe et al., 2000).

Another significant change conferred by *C*Las infection was with respect to amino acid metabolism. At the transcriptomic level, the metabolism pathways of glycine, serine, threonine, tryptophan, phenylalanine, tyrosine, and glutathione were significantly affected (Supplementary Table S5). Genomic comparisons of *C*Las with cultivable *L*. *crescens* revealed a lack of genes related to the production of fatty acids and aromatic amino acids (phenylalanine, tryptophan, and tyrosine) (Duan et al., 2009; Tyler et al., 2009; Lai et al., 2016). This suggests that *C*Las must rely on either the host plant or the insect vector for these essential nutrients, and therefore they may use these amino acids by modulating the corresponding metabolism pathways in *D. citri*. The metabolomic analysis also revealed the significant change in the tryptophan metabolism pathway (Figure 4). Moreover, *C*Las infection elevated the abundance of several aliphatic amino acids (glycine, L-asparagine, and beta-alanine), the metabolism of which is closely associated with the fatty acid metabolism.

Our results showed that *C*Las-infected *D. citri* tended to be enriched in fatty acids (Supplementary Table S6), which is in accordance with the results of previous studies (Killiny et al., 2017; Killiny and Jones, 2018). The increased abundance of linoleic acid was also observed in *C*Las-infected *D*. *citri* nymphs (Killiny and Jones, 2018). In contrast, the abundance of some fatty amides, derivatives of fatty acids, was significantly reduced. This means that *C*Las infection promotes the accumulation of fatty acids in *D. citri* through the modulation of fatty acid biosynthesis and the reduced transformation of fatty acids to the respective derivatives. The increasing abundance of fatty acids may also occur because of their important roles in the innate immunity of insects (Stanley et al., 2012). Remarkably, we observed the upregulation of lipid transporter activity and the significant overexpression of almost all vitellogenin genes (Supplementary Tables S3, S4). Vitellogenin is a lipoprotein with functions in insect fecundity and immunity, and it is synthesized in the fat body and secreted into the hemolymph (Arrese and Soulages, 2010). Vitellogenin plays an important nutritional role during vitellogenesis (Tufail and Takeda, 2008) and has been shown to function in insect immunity by binding to pathogen lipopolysaccharides and peptidoglycans (Li et al., 2009). Previous proteomic data have shown that *C*Las infection causes the upregulation of vitellogenin proteins in the *D. citri* hemolymph, gut, or whole body (Ramsey et al., 2017; Kruse et al., 2017, 2018). Our finding further supports the upregulation of vitellogenin in the *C*Las-infected *D. citri*, and this is in accordance with a previous report that showed the increasing reproductive fitness in *C*Las-infected *D. citri* (Pelz-Stelinski and Killiny, 2016).

The transcriptomic results also suggested that *C*Las modulated the cofactor and vitamin metabolism in *D. citri* (Figures 3B, 4C). Pantothenate, the starting compound in CoA biosynthesis, was found to be significantly reduced by *C*Las infection. This may result from the interaction between *C*Las metabolism and host proteins (Ramsey et al., 2017). Our study also indicated the decrease in riboflavin in *C*Las-infected insects. Riboflavin provisioning has been previously reported in bacteria–insect endosymbiotic relationships (Moriyama et al., 2015; Ju et al., 2020). Therefore, the observed reduction in riboflavin may result from the impact of *C*Las on other symbionts that confer fitness in riboflavin provisioning to *D. citri*, as *C*Las infection not only modulates host immunity but is also related to other microbes in the host insect (Ramsey et al., 2017; Kruse et al., 2018). In fact, bacteria benefiting from the host insect’s cofactor and vitamin biosynthesis is a common phenomenon (Bauer et al., 2014; Salem et al., 2014; Ju et al., 2020), and this may also explain the potential downregulation of folate biosynthesis and retinol metabolism pathways inferred from the transcriptomic analysis herein shown (Supplementary Table S5).

## Conclusion

In conclusion, by using transcriptomic and metabolomic analyses, we identified the genes and metabolites that differentially expressed between *C*Las-infected and non-infected *D. citri*. The integrated analysis of these two omics data revealed that *C*Las infection had a remarkable impact on pathways associated with the metabolism of amino acids, lipids, and cofactors. The present study provides a foundation for further investigation on the mechanisms of *C*Las*–D. citri* interaction.

## Data Availability Statement

The datasets presented in this study can be found in online repositories. The names of the repository/repositories and accession number(s) can be found in the article/Supplementary Material.

## Author Contributions

KL, RP, and QH conceived and designed the study and wrote the manuscript. JH, ZG, and MZ contributed to materials. KL and JH performed the experiments. RP performed the data analysis. All authors contributed to the article and approved the submitted version.

## Conflict of Interest

The authors declare that the research was conducted in the absence of any commercial or financial relationships that could be construed as a potential conflict of interest.

## Footnotes

1 www.metaboanalyst.ca/

## Supplementary Material

Supplementary Figure 1 The correlation of the RNA-seq datasets from *C*Las-infected and non-infected *D. citri*. The correlation was calculated according to Spearman’s rank correlation coefficient **(A)** and principal component analysis **(B)**.

Supplementary Figure 2 Orthogonal projections tolatent structures- discriminant analysis (OPLS-DA) plots of metabolite composition of samples from *C*Las-infected and non-infected *D. citri*. Each symbol represents a sample, different symbol shapes denote different groups. The circle line indicates the 95% confidence interval (Hotelling’s T-squared ellipse).

Supplementary Figure 3 Pathways of Glycerophospholipid metabolism **(A)** and Arachidonic acid metabolism **(B)** affected by *C*Las infection. Red indicates that the gene/compound is up-regulated, blue indicates that the gene/compound is down-regulated.

Supplementary Table 1 The sequences of primers used in this study.

Supplementary Table 2 The summary of RNA-seq datasets.

Supplementary Table 3 The expression statistics of all genes obtained by RNA-seq.

Supplementary Table 4 Results of the GO enrichment analysis for the DEGs.

Supplementary Table 5 Results of the KEGG pathway enrichment analysis for the DEGs.

Supplementary Table 6 The information of compounds identified by the metabolome analysis.

Supplementary Table 7 The pathways affected by the differential metabolites.

Supplementary Table 8 The overlapped pathways identified from transcriptome and metabolome analysis.
