# Supplementary figures and images for: Transcriptomic and Metabolomic Analyses of Diaphorina citri Kuwayama Infected and Non-infected With Candidatus Liberibacter Asiaticus
Source: Front Physiol. 2021 Feb 24;11:630037. doi: 10.3389/fphys.2020.630037 (PMC7943627; doi:10.3389/fphys.2020.630037)

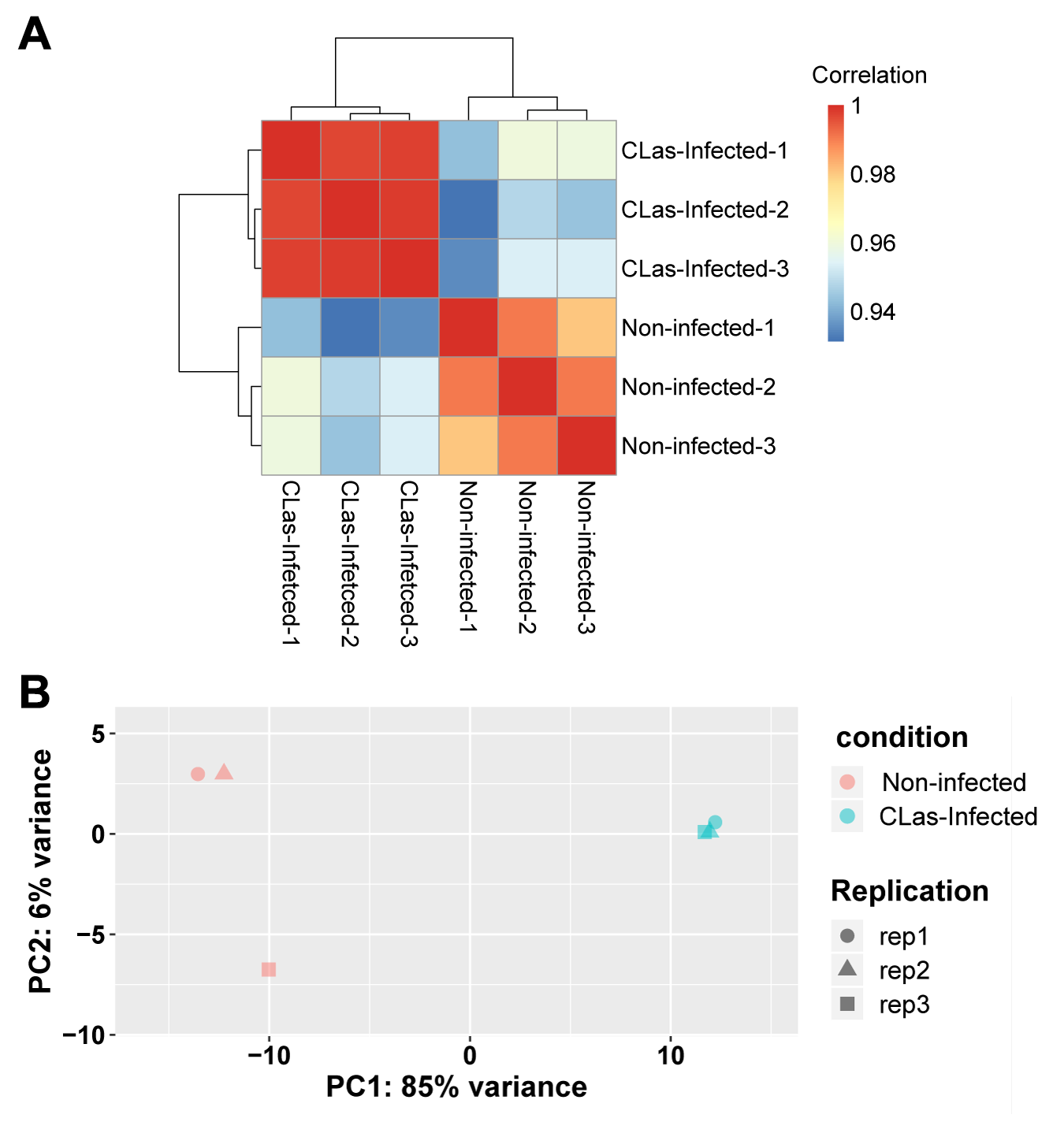

Supplement: Supplementary Figure 1 — The correlation of the RNA-seq datasets from CLas-infected and non-infected D. citri. The correlation was calculated according to Spearman’s rank correlation coefficient (A) and principal component analysis (B). [file Image_1.TIF]

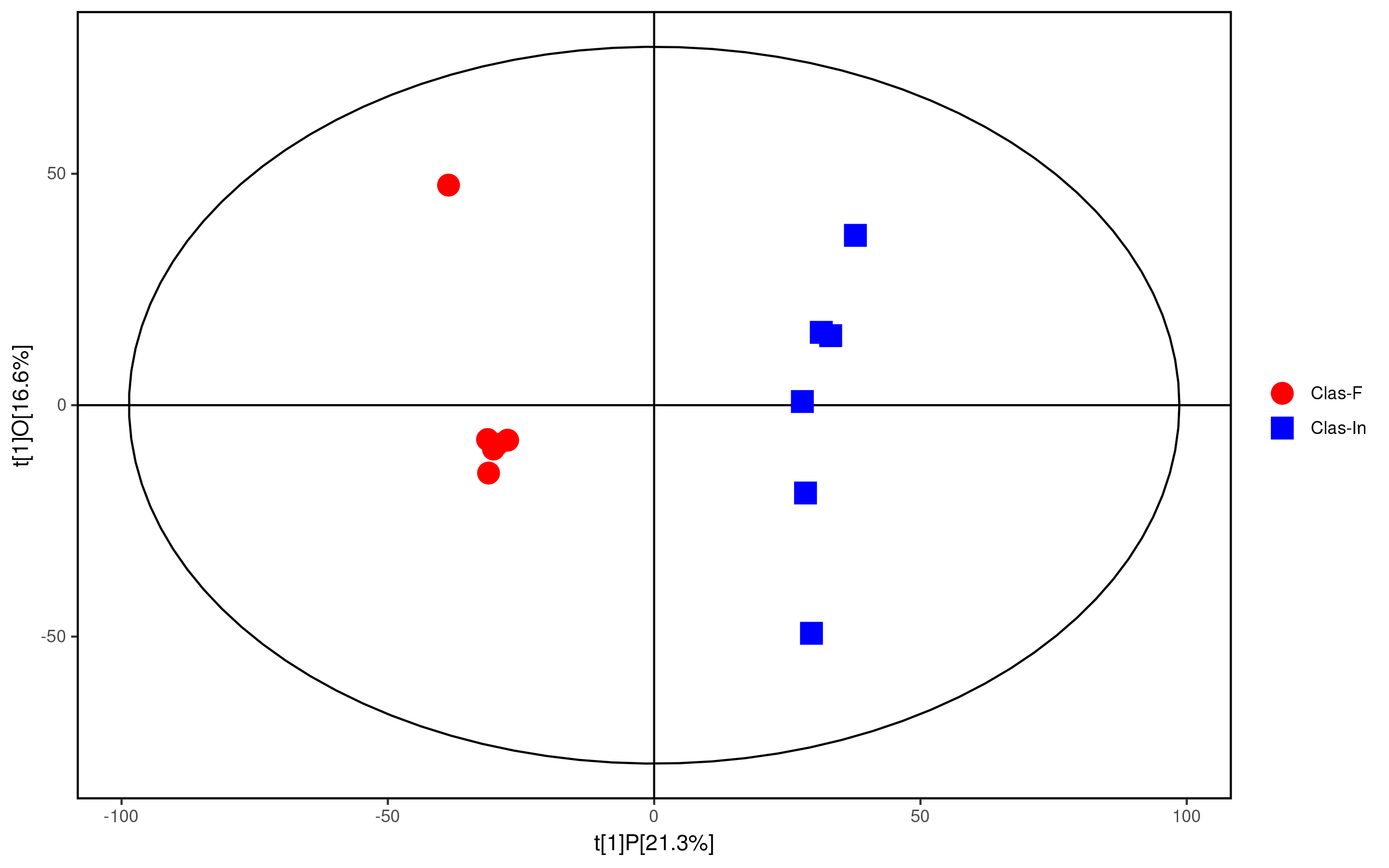

Supplement: Supplementary Figure 2 — Orthogonal projections tolatent structures- discriminant analysis (OPLS-DA) plots of metabolite composition of samples from CLas-infected and non-infected D. citri. Each symbol represents a sample, different symbol shapes denote different groups. The circle line indicates the 95% confidence interval (Hotelling’s T-squared ellipse). [file Image_2.TIF]

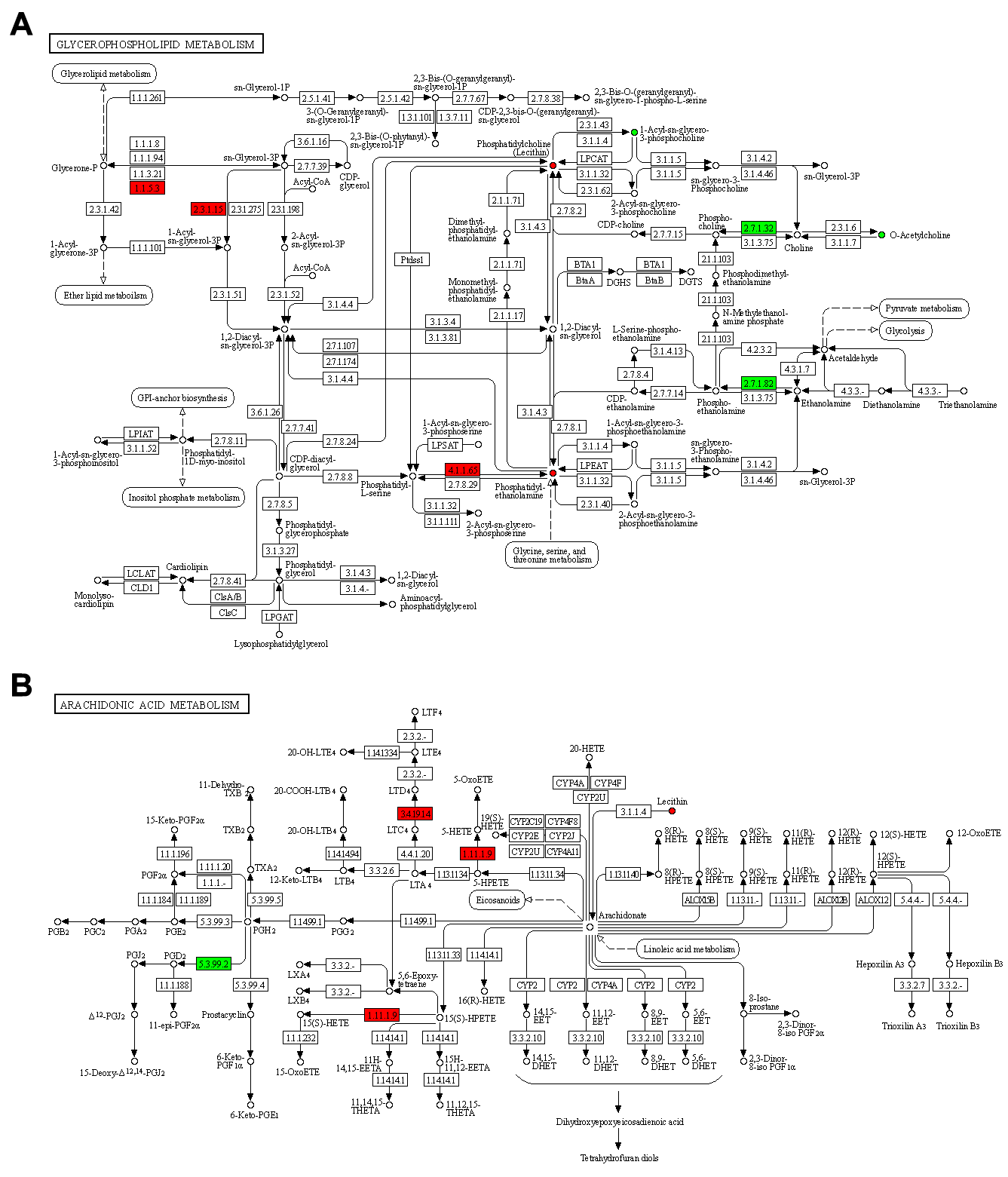

Supplement: Supplementary Figure 3 — Pathways of Glycerophospholipid metabolism (A) and Arachidonic acid metabolism (B) affected by CLas infection. Red indicates that the gene/compound is up-regulated, blue indicates that the gene/compound is down-regulated. [file Image_3.TIF]
